# Development and usability testing of a very brief intervention for personalised cancer risk assessment to promote behaviour change in primary care using normalisation process theory

**DOI:** 10.1017/S146342361900080X

**Published:** 2020-01-14

**Authors:** Katie Mills, Simon J. Griffin, Stephen Sutton, Juliet A. Usher-Smith

**Affiliations:** 1 Research Associate, The Primary Care Unit, Department of Public Health and Primary Care, University of Cambridge School of Clinical Medicine, Cambridge, UK; 2 Professor of General Practice, The Primary Care Unit, Department of Public Health and Primary Care, University of Cambridge School of Clinical Medicine, Cambridge, UK; 3 Professor of Behavioural Science, The Primary Care Unit, Department of Public Health and Primary Care, University of Cambridge School of Clinical Medicine, Cambridge, UK; 4 Clinical Senior Research Associate, The Primary Care Unit, Department of Public Health and Primary Care, University of Cambridge School of Clinical Medicine, Cambridge, UK

**Keywords:** intervention development, cancer risk, behaviour change, primary care

## Abstract

**Background:**

Cancer is the second leading cause of death worldwide. Lifestyle choices play an important role in the aetiology of cancer with up to 4 in 10 cases potentially preventable. Interventions delivered by healthcare professionals (HCPs) that incorporate risk information have the potential to promote behaviour change. Our aim was to develop a very brief intervention incorporating cancer risk, which could be implemented within primary care.

**Methods:**

Guided by normalisation process theory (NPT), we developed a prototype intervention using literature reviews, consultation with patient and public representatives and pilot work with patients and HCPs. We conducted focus groups and interviews with 65 HCPs involved in delivering prevention activities. Findings were used to refine the intervention before 22 HCPs completed an online usability test and provided further feedback via a questionnaire incorporating a modified version of the NoMAD checklist.

**Results:**

The intervention included a website where individuals could provide information on lifestyle risk factors view their estimated 10-year risk of developing one or more of the five most common preventable cancers and access lifestyle advice incorporating behaviour change techniques. Changes incorporated from feedback from the focus groups and interviews included signposting to local services and websites, simplified wording and labelling of risk information. In the usability testing, all participants felt it would be easy to collect the risk information. Ninety-one percent felt the intervention would enable discussion about cancer risk and believed it had potential to be easily integrated into National Health Service (NHS) Health Checks. However, only 36% agreed it could be delivered within 5 min.

**Conclusions:**

With the use of NPT, we developed a very brief intervention that is acceptable to HCPs in primary care and could be potentially integrated into NHS Health Checks. However, further work is needed to assess its feasibility and potential effectiveness.

## Background

Cancer is now the second leading cause of death worldwide (World Health Organisation, 2018). Approximately 4 in 10 cases are thought to be preventable through lifestyle change. The importance of prevention has been highlighted in both the Academy of Medical Sciences ‘Improving the health of the public by 2040’ report (The Academy of Medical Sciences, 2016) and in the National Health Service (NHS) ‘Five Year Forward View’, in which the sustainability of the health system is described as being dependent on ‘radical upgrade in prevention and public health’(National Health Service, 2014).

As described in those reports, achieving this change is likely to require interventions targeted at both the population and individual level. Primary care provides an ideal platform from which to deliver individual-level interventions. Not only does primary care provide over 300 million patient consultations each year in England alone (National Health Service, 2014), but it is also the site in which many other prevention programmes, including the NHS Health Check and Diabetes Prevention programmes in England (NHS England, 2019), are already delivered.

A common component of many prevention programmes is the estimation and communication of risk of disease. The evidence for behaviour change following provision of risk information in general is limited (Usher-Smith *et al.*, 2015; Hollands *et al.*, 2016; French *et al.*, 2017). However, a recent systematic review of randomised trials showed that interventions incorporating personalised non-genetic cancer risk information were associated with increased odds of remaining a former smoker in those who had recently quit smoking and increased sun protection habits, skin self-examination and breast examination (Usher-Smith *et al.*, 2018b). Behaviour change interventions incorporated within breast and colorectal cancer screening programmes have also achieved significant reductions in multiple risk factors (Emmons *et al.*, 2005; Anderson, Craigie *et al.*, 2014; Anderson, Macleod *et al.*, 2014). Provision of cancer-specific risk information alongside lifestyle advice at an individual level within the context of primary care may therefore support population-level interventions to promote behaviour change.

As with all healthcare professional (HCP)-led interventions, success depends on the engagement of those delivering the intervention. While studies have confirmed that HCPs in primary care consider prevention an important part of their role, delivering prevention activities is considered difficult for many and is not routinely conducted (Brotons *et al.*, 2005; Noordman *et al.*, 2010; McIlfatrick *et al.*, 2013; Usher-smith *et al.*, 2017a). Barriers identified include lack of time (Brotons *et al.*, 2005; McIlfatrick *et al.*, 2013; Usher-smith *et al.*, 2017a), training (McIlfatrick *et al.*, 2014; Usher-smith *et al.*, 2017a) and availability of clear resources for patients (Usher-smith *et al.*, 2017a). To address these barriers and other factors contributing to the ‘implementation gap’ between research and practice (Olswang and Prelock, 2015), a number of theories have been developed. One is normalisation process theory (NPT), which provides a framework for understanding how and whether complex interventions become routinely embedded in healthcare practice (May *et al.*, 2009). It focuses on the work that individuals and groups do to enable an intervention to become normalised and includes four components: coherence (sense-making), cognitive participation (engagement), collective action (enactment) and reflective monitoring (appraisal). It has been widely used to successfully retrospectively analyse the implementation of interventions (McEvoy *et al.*, 2014; May *et al.*, 2018) and has also been proposed as a tool to be applied prospectively to raise awareness about facilitators and barriers to successful implementation (Murray *et al.*, 2010). Used in this way, it can act as a ‘sensitising tool’ (Murray *et al.*, 2010) to encourage thinking through issues around implementation when designing interventions.

The Medical Research Council guidance for development and evaluation of complex interventions (Craig *et al.*, 2008) and The National Institute for Health and Care Excellence Public Health guidance for behaviour change interventions (National Institute for Health and Care Excellence, 2007) also emphasise the importance of the early phases of intervention development and the need to ensure that interventions build on the skills, talents and capacity of HCPs and are consistent with other local and national interventions and programmes (National Institute for Health and Care Excellence, 2007).

We aimed to use NPT alongside HCPs currently working within primary care to guide the development of a very brief risk-based intervention that could be used within primary care to support patients to make lifestyle changes to prevent cancer.

## Methods

**Figure 1.**
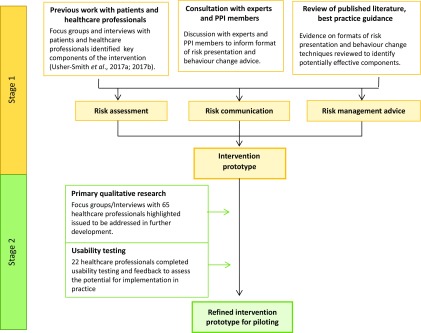
Development and testing process of the prototype intervention.

The overall process for developing and testing the intervention is summarised in Figure 1.

### Stage 1: Development of a prototype intervention

To guide the initial format of the prototype and how it might fit within the primary care context, we began by considering the four components within each of the core constructs within NPT: coherence; cognitive participation; collective action; and reflexive monitoring. Coherence refers to the sense-making by participants either individually or collectively when faced with the implementation of a new set of practices; cognitive participation relates to participant understanding and engagement with the new set of practices within their current roles; collective action considers the capacity and support needed for the incorporation of the new practices into existing procedures; and reflexive monitoring describes participant appraisal, evaluation and monitoring of the impact of the new practices on themselves and their working roles (Normalisation process theory constructs, 2019). Guided by the questions within the NPT toolkit (Normalisation process theory-NPT toolkit, 2019), we considered the application of each of these constructs to the intervention in turn.

To be consistent with the overall structure of other local and national risk communication-based interventions currently in use in primary care, such as NHS Health Checks (Public Health England, 2016), we considered the intervention in three parts:

- Risk assessment – a risk assessment tool to enable collection of diet and other lifestyle risk factors for cancer, either independently or with a HCP.
- Risk communication – a web-based tool to display the estimated risk of developing one or more cancers based on potentially modifiable lifestyle risk factors.
- Risk management advice – the opportunity to discuss behaviour change using evidence-based information on diet and lifestyle risk factors and signposting to existing services.

#### Development of risk assessment

To facilitate implementation, we chose to develop an online lifestyle-based risk assessment with an integrated data collection tool that required only simple data on lifestyle factors that could be collected by HCPs in a few minutes or self-completed by patients either in the waiting room or online prior to their appointment. To enable individuals to see the effect of lifestyle on multiple cancers, we chose to estimate the 10-year risk of developing one of the five commonest preventable cancers amongst men and women in the UK. These are lung, colorectal, bladder, kidney and oesophageal cancer for men; and breast, lung, colorectal, endometrial and kidney cancer for women.

The development and assessment of the performance of these lifestyle-based risk assessments is discussed in detail in a separate paper (Usher-Smith *et al.*, 2018a). In summary, established lifestyle risk factors from the European Code against Cancer (Anderson *et al.*, 2015; Leitzmann *et al.*, 2015; Leon *et al.*, 2015; Norat *et al.*, 2015; Scoccianti *et al.*, 2015) and estimates of relative risks from meta-analyses of observational studies were used to calculate an individuals’ risks of developing one or more of the five cancers relative to a recommended lifestyle. Mean values for risk factors from the Health Survey for England 2005 (available from: https://digital.nhs.uk/data-and-information/publications/statistical/health-survey-for-england) and the National Diet and Nutrition survey years 1–4 (2008/12) (available from: https://discover.ukdataservice.ac.uk/catalogue/?sn=6533)and mean 10-year estimated absolute risks from routinely available sources (Office for National Statistics, 2015a; 2015b) were then used to calculate the estimated absolute risk of developing one or more of the cancers over a 10-year period. The performance of the risk assessment was then validated using data from 23 768 participants (12 828 women and 10 940 men) in the EPIC-Norfolk cohort (Day *et al.*, 1999) who had at least 10-year follow-up and data for all risk factors and no previous history of diagnosis or any of the chosen cancers at baseline.

#### Development of risk communication

To enable communication of the risk to participants, we developed a web-based tool integrated within the Gorilla.sc research platform (www.gorilla.sc/about). In order to choose the format(s) in which to present the risk, we conducted several steps. This included: looking back at pilot work with members of the public in which they had been presented with their risk of individual cancers in four different ways and focus groups with HCPs within primary care which have been reported separately (Usher-smith *et al.*, 2017a; 2017b); a scoping review of literature published up to February 2017 that reported on the effectiveness and patient preferences of different risk presentation formats used in cardiovascular disease (CVD) and cancer (Fortin *et al.*, 2001; Julian-Reynier *et al.*, 2003; Kirby & Machen, 2009; Sheridan *et al.*, 2009; Hill *et al.*, 2010; Waldron *et al.*, 2011; Dorval *et al.*, 2013) reference to best practice guidance for communication of risk (Lipkus, 2007; Trevena *et al.*, 2013; Zipkin *et al.*, 2014); and discussions with patient and public representatives and experts in the field.

#### Development of risk management advice

Given the known challenges to achieving behaviour change and the evidence from systematic reviews of the limitations of risk provision alone (Brindle *et al.*, 2006; Usher-Smith *et al.*, 2015; French *et al.*, 2017), we set out to incorporate established behaviour change techniques (BCTs) into the intervention, within the consultation with the HCP, on the website and as a leaflet to be given to patients after the consultation. We began with the BCTs within the BCT Taxonomy (v1) (Michie *et al.*, 2013) which were judged appropriate by a consensus of experts in behaviour change and most frequently used for enablement and education interventions (Michie *et al.*, 2014). From that list, we then used the following three criteria to select which to include in the intervention:

1. Evidence for effectiveness of BCTs in this context
2. Relevance to the context, that is, BCTs that could be used within face-to-face interventions within primary care to promote lifestyle change to reduce future risk of cancer
3. Feasibility, that is, can be delivered by nurses/healthcare assistants within 5 min in primary care

To identify evidence for the first of these criteria, we performed a scoping review of the literature. This included searching online bibliographic databases in May 2017 to identify systematic reviews and meta-analyses published in English and reporting the effectiveness of the inclusion of individual BCTs on behaviour change. We then also screened the reference lists of identified papers for other relevant reviews.

### Stage 2: Refinement and testing of prototype intervention

#### Focus groups and interviews with healthcare professionals

To enable us to demonstrate the prototype intervention and receive direct feedback from key stakeholders, we conducted focus groups and face-to-face interviews with HCPs involved in delivering preventive healthcare across the East of England and London between June and August 2017. Approvals were obtained from the University of Cambridge Psychology ethics committee (Ref: PRE.2017.043) and the Health Research Authority (HRA) (Ref: 17/HRA/1948).

##### Participants and recruitment

To recruit HCPs currently working within general practice, letters of invitation and the study information leaflet were emailed to all general practitioners (GPs), practice nurses and healthcare assistants across Cambridge and Peterborough by the local Clinical Lead for the NHS Health Check programme. Those interested in taking part were invited to contact the research team directly. HCPs working within three health service commissioned providers of lifestyle advice were similarly emailed a letter of invitation along with the study information leaflet by their manager and invited to attend one of several planned focus groups. The local NIHR Clinical Research Network also provided assistance in the recruitment of HCPs from local general practices.

##### Data collection

All focus groups and interviews were held at the participants’ place of work and were led by a non-clinical researcher experienced in qualitative research (KM). Each lasted between 20 and 60 min. Written consent was obtained from all participants. Each focus group began with a presentation showing screenshots of the questions used to collect the risk factor information, presentation of risk and web-based lifestyle advice. Copies of the behaviour change leaflet were then handed out for participants to read. The discussions that followed were informed by a topic schedule (Appendix 1) which incorporated the first three NPT constructs (coherence, cognitive participation and collective action). We chose not to include the fourth construct, reflexive monitoring, as this relates to how individuals and groups assess how the intervention affects them in practice, and we felt that this would be difficult for participants at this stage to consider. Within focus groups, we also explored views of the participants on the overall format, content and length of the prototype intervention, as well as any barriers and facilitators to its incorporation into practice.

##### Analysis

The focus groups and interviews were audio-recorded and then transcribed verbatim and analysed using an iterative process which started near the beginning of data collection. Throughout this process, the qualitative data were fine-coded by one researcher (KM) with the aid of NVivo software (QSR International, version 11). Emergent themes were identified using thematic analysis (Braun and Clarke, 2006) and then discussed amongst the wider research team and used to refine the prototype intervention.

#### Usability testing and feedback from healthcare professionals

After further refinement of the intervention based on the findings from the focus groups and interviews, we developed the web-based intervention and invited HCPs to trial the website and provide feedback on its usability and the intervention as a whole.

##### Participants and recruitment

All participants who had taken part in a focus group or interview and who had provided a valid email address were sent an email with a link to the intervention website. A unique study ID was included in each email that enabled the participants to log in and work through the entire intervention as if they were delivering it in practice. This included collection of information about lifestyle risk factors, presentation of the estimated risk, setting target values and seeing the impact of those changes on the estimated risk, and then viewing all the pages of the behaviour change leaflet. They were then automatically directed to an electronic questionnaire.

##### Data collection

The electronic questionnaire was in two parts, Appendix 2. The first asked participants about the usability of the website and the clarity of the information provided. The second focused on the potential for the intervention to be incorporated into practice with questions covering the first three components of NPT adapted from the NoMAD checklist (Finch *et al.*, 2013) in line with guidance from the NPT website (Normalisation process theory, 2019). In the second section, we also included specific questions about the anticipated duration of the intervention and the potential for it to be incorporated within NHS Health Checks, routine consultations, chronic disease reviews and lifestyle advice consultations.

##### Analysis

Data from the questionnaire were analysed descriptively and are presented as frequencies and means (±standard deviation, SD). Agreement with statements from the NoMAD checklist was converted into a five-point scale ranging from 1 (Strongly disagree) to 5 (Strongly agree). Those selecting the option ‘Not applicable to my role’ were treated as missing data for that question.

## Results

### Stage 1: Development of a prototype intervention

Table 1 shows how each of the four components of the four core constructs within NPT were used to inform decisions about the overall concept, content and delivery of the intervention. Particular considerations included making the intervention simple to describe to patients; ensuring intuitive navigation to minimise training requirements; and designing it to fit within current prevention activities within primary care such as NHS Health Checks.

**Table 1.** Applying NPT to development of the intervention

NPT construct and components	Description^a^	Considerations for prototype intervention design/delivery
*Coherence*		
Differentiation	Whether the intervention is easy to describe to participants and whether they can appreciate how it differs or is clearly distinct from current ways of working	Make the intervention simple to describe, with visual elements for ease of comprehension and completion. Host on a standalone website so not to interfere with current software in practice.
Communal specification	Whether participants have or are able to build a shared understanding of the aims, objectives and expected outcomes of the proposed intervention	Align the aims, objectives and expected outcomes (ie, to promote behaviour change to prevent disease) with those for NHS Health Checks and other prevention activities in primary care and make these clear in the training for the intervention.
Individual specification	Whether individual participants have or are able to make sense of the work – specific tasks and responsibilities – the proposed intervention would create for them	Provide clear guidance and training on delivery of the intervention. Limit the additional work delivery will create for individuals by developing a leaflet and website that patients can refer back to after the consultation.
Internalisation	Whether participants have or are able to easily grasp the potential value, benefits and importance of the intervention	Design to fit initially within current prevention activities within primary care, such as NHS Health Checks and chronic disease reviews.
Cognitive participation		
Initiation	Whether or not key individuals are able and willing to get others involved in the new practice	Engage with both those delivering the intervention and their managers/employers and include of clear justification for the importance of focusing on behaviour change for cancer prevention and parallels with other existing activities within the practice.
Legitimation	Whether or not participants believe it is right for them to be involved, and that they can make a contribution to the implementation work	Distinguish between the benefit of providing risk information and the role of face-to-face communication within the intervention to enable HCPs to see the added value they provide.
Enrolment	The capacity and willingness or participants to organise themselves in order to collectively contribute to the work involved in the new practice	Structure the intervention to minimise the need for re-organisation or additional capacity and do not attempt to develop a standardised process for delivery to allow HCPs to adapt it to different consultations and patient groups.
Activation	The capacity and willingness of participants to collectively define the actions and procedures needs to keep the new practice going	Work with HCPs throughout the implementation stage to help them adapt the intervention to suit their local context and provide regular feedback.
Collective action		
Interactional workability	Whether people are able to enact the intervention and operationalise its components in practice	Consideration for the length of time needed to deliver the intervention to minimise impact on current consultation length.
Relational integration	Whether people maintain trust in the intervention and in each other	Ensure it fits with the overall objectives and current prevention activities such as NHS Health Checks.
Skill set workability	Whether the work required by the intervention is seen to be parcelled out to participants with the right mix of skills and training to do it	Design of the intervention to be simple and navigation intuitive to minimise staff requirement for training before use.
Contextual integration	Whether the intervention is supported by management and other stakeholders, policy, money and material resources	Inclusion of managers in focus groups/interviews and usability testing, to obtain their views on aspects of the prototype design and its delivery to establish potential resource and support constraints.
Reflexive monitoring		
Systematisation	The collection of information in a variety of ways to seek how effective and useful for participants in any set of practices may seek to determine how effective and useful it is for them and for others, and this involves the work of collecting information in a variety of ways	Collection of data from key individuals in a variety of formats including both qualitative and quantitative methodologies.
Communal appraisal	Whether participants work together, formally or informally to evaluate a set of practices.	Provide participants with the opportunity to adapt the intervention collectively and evaluate the potential impact of the intervention in their own setting.
Individual appraisal	Whether participants in a new set of practices also work experimentally as individuals to appraise its effect on them and the contexts in which they are set. From this individuals express their personal relationships with the new set of practices.	Provide opportunities for key individuals to provide feedback in the planning and development of the intervention to facilitate design for incorporation into normal practices.
Reconfiguration	Appraisal work by individuals or in groups lead to attempts to modify practices.	For potential to adapt after initial usability testing.

NPT = normalisation process theory; NHS = National Health Service; HCPs = Healthcare professionals.

a From Normalisation Process Theory Toolkit.

#### Development of risk communication

Table 2 details the key findings which we considered when choosing the format in which to communicate the risk. In addition to the previously published pilot work (Usher-Smith *et al.*, 2017a) and best practice guidance (Fortin *et al.*, 2001; Lipkus, 2007; Waldron *et al.*, 2011), we identified seven studies (13–18). Key considerations included: the appropriate use of a colour scale to demonstrate the level of risk; inclusion of relative risk to promote behaviour change; a 10-year risk estimate to align with current CVD risk estimates; and the ability to change modifiable risk factors and view their effect on overall risk estimate. The chosen format for risk presentation was a bar graph displaying a 10-year risk estimate. This included colour shading to communicate the level of risk on a scale from green to red. The graph axis described an individual’s risk level as the number of times higher than that of a person following all of the recommended lifestyle guidance. The risk presentation displays this as an additional bar for reference. To aid interpretation, the percentage value of the risk level can also be viewed. On setting new target values for lifestyle changes, the bar graphs display an additional level of risk to visualise the consequent potential risk reduction. On completion, the bar graph communicates three levels of risk: (1) current, (2) potential future risk after making lifestyle improvements and (3) the risk if following all of the recommended lifestyle guidance.

**Table 2.** Evidence used to inform choice of format of risk presentation

Finding	Inclusion in prototype intervention design/delivery
*Pilot work with members of the public and healthcare professionals*	
When presented in colour, the colour was often more important than the number and dominated their interpretation (Usher-Smith *et al.*, 2017b).	Inclusion of colour in risk presentation while ensuring that the colour scheme reflects current evidence/expert opinion.
Being able to see the impact of changes in lifestyle on their risk was helpful. This included the effect of small changes (increasing fruit and vegetable consumption by one portion per day rather than meeting the requirement of five portions per day). Some also wanted to be able to see the benefits they were already achieving through their current lifestyle (Usher-Smith *et al.*, 2017b).	Incorporation of ways to demonstrate continuous change, both positive and negative, for each modifiable factor.
The first reaction of almost all when presented with their 10-year risk of an individual cancer was that it was low and not concerning, with views on what constituted a high risk ranging widely, from 0.5% to 60 %. As a result, reductions in risk were not always motivating – the risks were considered low and differences small (Usher-Smith *et al.*, 2017b).	Provision of combined risk of multiple cancers.
*Review of published literature and best practice guidance*	
Numerical presentation of risk as opposed to simple risk categories (moderate, high, low) appears to lead to more accurate risk perception (Waldron *et al.*, 2011) and when investigating only the patient’s preferences towards cancer risk communication, the majority of the British women and 50% of the Australian women expressed their preferences for quantitative risk information (Julian-Reynier *et al.*, 2003).	Inclusion of option to see risk as a percentage.
There were strong objections to the word ‘absolute’, which was seen as ambiguous. For many participants it conveyed that the risk score was ‘conclusive’, or in some way ‘definite’ that a person would suffer a cardiovascular event rather than a probability (Kirby & Machen, 2009; Hill *et al.*, 2010).	Avoidance of the term ‘absolute risk’ and clarity throughout that risks are estimates and apply to people with the same characteristics as the individual rather than the individual person.
People need comparisons between the probabilities of different risks in order to be able to interpret absolute risk information (Julian-Reynier *et al.*, 2003; Hill *et al.*, 2010).	Provision of relative risk in addition to absolute risk information and comparison to individuals with a recommended lifestyle.
Presenting relative risk as number alone has been criticised as many participants did not know how to translate 2.3 times in absolute terms (Dorval *et al.*, 2013) or because it was ‘too alarming because the risks appeared bigger’ (Fortin et al., 2001).	Inclusion of option to see risk as an absolute percentage and comparison with individual with recommended lifestyle
Treatment decisions are sensitive to the way a treatment’s effectiveness is presented. The relative risk reduction format appears to encourage the treatment the most and number needed to treat format leads to the least acceptance (Waldron *et al.*, 2011).	Presentation of relative risk to encourage behaviour change.
Shorter timeframes (less than 10 years) may lead to more accurate risk perceptions and increased intention to change behaviour, than 10-year risk or longer, especially for older patients (Waldron *et al.*, 2011). Some participants thought 10 years was too remote (Hill *et al.*, 2010).	Decision made to present 10-year risk to be consistent with cardiovascular disease within primary care.
Display of risk information visually can enhance understanding compared with written information alone, particularly amongst those with low numeracy (Lipkus, 2007)	Display risk information with a simple visual for ease of understanding.
Graphical formats are perceived as helpful (Hill *et al.*, 2010) but one format does not fit all (Dorval *et al.*, 2013). Several formats were reported as confusing, such as line graphs, and icons, particularly those with larger numbers (Hill *et al.*, 2010).	Inclusion of graphical presentation but avoid line graphs and icons.
People found formats which combined information helpful, such as colour, effect of changing behaviour on risk or comparison with a healthy older person (Hill *et al.*, 2010).	Inclusion of colour, effect of changing behaviour and comparison to individual with a recommended lifestyle.
Provision of feedback from the consultation to the counselee appears to be welcomed and the interest in other tools that complement the consultation has been pointed out (eg, leaflets, CDs and other media to promote self-help) including the tailored print communication through a personal letter summarising the consultation for the counselee (Sheridan *et al.*, 2009).	Inclusion of option to print a tailored information sheet summarising the risk assessment.
Several explained they might take their risk more seriously if they knew exactly what the calculation is based on and how the numbers affect the final percentage (Sheridan *et al.*, 2009).	Provision for individuals to change all the modifiable factors to see how that changes the final risk estimate and provided information on the development of the risk score as additional information.
*Consultation with experts and PPI members*	
To enable understanding of risk, incorporation of colour into the risk presentation. For this to be of use, it must have meaning.	Inclusion of a colour scale from green to red to demonstrate level of risk where green corresponds to a relative risk of 1 and then the colour changes gradually to be orange at a relative risk of 2 and then to red at a relative risk of 4
Use of relative risk is acceptable in the context of this study; however, this must be made clear to the recipient.	Clarity throughout that risks are estimates and apply to people with the same characteristics as the individual rather than the individual person.

CD = compact disc; PPI = patient and public involvement.

#### Development of risk management advice

From the 93 BCTs within the BCT Taxonomy (v1) (Michie *et al.*, 2013), 58 were judged appropriate by a consensus of experts in behaviour change and most frequently used for enablement and education interventions (Michie *et al.*, 2014). We then identified four systematic reviews (Michie *et al.*, 2009; Lara *et al.*, 2014; McDermott *et al.*, 2016; Samdal *et al.*, 2017) addressing which of these BCTs are most effective in the context of changes in physical activity and diet. To our knowledge, no systematic reviews have reported the effectiveness of BCTs in the context of alcohol consumption and smoking. Overall, the evidence for effectiveness of the BCTs was mixed. However, the reviews did identify a number of BCTs associated with intention and behaviour change. In the study by Lara *et al.* (2014), the BCTs ‘plan for social support’ and ‘goal setting (outcome)’ were reported to make clinically important improvements in fruit and vegetable consumption (Lara *et al.*, 2014). McDermott *et al.* (2016) reported that no BCTs were associated with significant positive effects on behaviour. However, they did identify that there was a significant positive association of intention with the BCT ‘provide information on the consequences of behaviour in general’ (McDermott *et al.*, 2016). Michie *et al.* (2009) reported that interventions designed to promote physical activity and healthy eating appear to be more effective if the BCT ‘self- monitoring’ and at least one of the four other self-regulatory techniques derived from control theory (Carver and Scheier, 1982) (‘prompt intention formation’, ‘prompt specific goal setting’, ‘provide feedback on performance’, ‘prompt review of behavioural goals’) were included (Michie *et al.*, 2009). Similarly, a more recent study by Samdal *et al.* (2017) described ‘self-monitoring of behaviour’ and ‘goal setting of behaviour’ as associated with a positive intention effect for both short- and long-term changes (Samdal *et al.*, 2017).

The reviews also identified BCTs negatively associated with change. For example, ‘exploring the pros and cons of behaviour change’ was reported as negatively associated with changes in diet and physical activity in overweight and obese adults (Samdal *et al.*, 2017), ‘relapse prevention/coping planning’ was associated with a negative change in intention (McDermott *et al.*, 2016), and ‘provide feedback on performance’ was reported to have a significant negative effect on behaviour (McDermott *et al.*, 2016). We, therefore, excluded these BCTs from our selection.

After assessing each of the remaining BCTs against our additional criteria of relevance to the context of primary care and practicability to deliver within a 5-min consultation, we selected 13 to include in the intervention (Table 3). These include social support (unspecified); goal setting (behaviour); goal setting (outcome); and self-monitoring of behaviour and, as described in Table 3, are incorporated within both the consultation itself and the written information provided as part of the intervention. For example, the website allows demonstration of the estimated cancer risk and impact of lifestyle change, and the behaviour change leaflet (appendix 3) includes generic advice on goal setting and support with signposting to local services and information on each of the lifestyle risk factors with details on their association with cancer, suggestions for lifestyle improvements and space to write goals. The prototype intervention, therefore, consisted of a website where on completion of a questionnaire on lifestyle cancer risk factors, a 10-year risk estimate, is presented as a coloured graded bar graph. Lifestyle improvements discussed supported by weblinks and paper copy of a behaviour change leaflet including signposting to local services, target values set for lifestyle risk factors entered onto the website and a target level of risk calculated to visualise potential risk reduction.

**Table 3.** Selection of behaviour change techniques

BCT^a^	Description	Evidence for effectiveness^b^	Relevant to context	Practical criteria	Inclusion in prototype intervention design/delivery
**Goal setting (behaviour)**	**Set or agree a goal defined in terms of the behaviour to be achieved**	**✓✓-**	✓	✓	**Statement in introduction about benefits of goal setting/action planning and examples and space to include their goals**
**Problem-solving**	**Analyse, or prompt the person to analyse, factors influencing the behaviour and generate or select strategies that include overcoming barriers and/or increasing facilitators**	**✓✓- ✗**	✓	✓	**Statement on support page of leaflet**
**Goal setting (outcome)**	**Set or agree a goal defined in terms of a positive outcome of wanted behaviour**	✓	✓	✓	**Statement in introduction about benefits of goal setting/action planning and examples and space to include their goals**
**Action planning**	**Prompt detailed planning of performance of the behaviour (must include at least one of context, frequency, duration and intensity)**	**-**	✓	✓	**Statement in introduction about benefits of goal setting/action planning and examples and space to include their goals**
Review behavioural goal(s)	Review behaviour goal(s) jointly with the person and consider modifying goal(s) or behaviour change strategy in light of achievement.	✓✓-	✓	✗	---
Review outcome goal(s)	Review outcome goal(s) jointly with the person and consider modifying goal(s) in light of achievement.		✓	✗	---
Feedback on behaviour	Monitor and provide information or evaluative feedback on performance of the behaviour.	✓	✓	✗	---
**Self-monitoring of behaviour**	**Establish a method for the person to monitor and record their behaviour(s) as part of a behaviour change strategy**	✓✓	✓	✓	**Statement in lifestyle advice page on physical activity, with reference to the use of pedometers for self-monitoring**
Feedback on outcome(s) of behaviour	Monitor and provide feedback on the outcome of performance of the behaviour.	✓	✓	✗	---
**Social support (unspecified)**	**Advise on, arrange or provide social support (eg, from friends, relatives, colleagues or staff) or non-contingent praise or reward for performance of the behaviour.**	**✓✓-**	✓	✓	**Statement in introduction describing how social support can be helpful to achieve changes in lifestyle with examples**
**Social support (practical)**	**Advise on, arrange or provide practical help (eg, from friends, relatives, colleagues or staff) for performance of the behaviour.**		✓	✓	
**Information about health consequences**	**Provide information (eg, written, verbal, visual) about health consequences of performing the behaviour**		✓	✓	**Risk of developing cancer with different lifestyles described verbally and visually with risk presentation**
**Information about social and environmental consequences**			✓	✓	**Statement on saving money on quitting smoking page of leaflet**
**Social comparison**	**Draw attention to others’ performance to allow comparison with the person’s own performance**	**-**	✓	✓	**Comparison to someone with a recommended lifestyle included in risk presentation**
Prompts/cues	Introduce or define environmental or social stimulus with the purpose of prompting or cueing the behaviour. The prompt or cue would normally occur at the time or place of performance.	-	✓	✗	---
Behavioural substitution	Prompt substitution of the unwanted behaviour with a wanted or neutral behaviour		✓	✗	---
**Habit formation**	**Prompt rehearsal and repetition of the behaviour in the same context repeatedly so that the context elicits the behaviour**		✓	✓	**Statement within introduction about habit formation and the fact that this can take several months to develop**
Habit reversal	Prompt rehearsal and repetition of an alternative behaviour to replace an unwanted habitual behaviour		✓	✗	---
Generalisation of a target behaviour	Advise to perform the wanted behaviour, which is already performed in a particular situation, in another situation		✓	✗	---
Graded tasks	Set easy-to-perform tasks, making them increasingly difficult, but achievable, until behaviour is performed.	✓-	✓	✗	---
**Credible source**	**Present verbal or visual communication from a credible source in favour of or against the behaviour**		✓	✓	**Reference to Cancer Research UK and University of Cambridge ‘experts’ with further resources at the end**
Pros and cons	Advise the person to identify and compare reasons for wanting and not wanting to change the behaviour.	✗	✓	✗	---
**Comparative imagining of future outcomes**	**Prompt or advise the imagining and comparing of future outcomes of changed versus unchanged behaviour**		✓	✓	**Opportunity to change lifestyle and see impact on risk and for people to revisit the website and amend their risk factors in the future**
Restructuring of environment			✗	✗	---
Avoidance/reducing exposure to cues for the behaviour	Advise on how to avoid exposure to specific social and contextual/physical cues for the behaviour, including changing daily or weekly routines		✓	✗	---
Adding objects to the environment		✓	✗	✗	---

BCT = behaviour change technique.

a Behaviour change techniques are ordered by the Taxonomy [7]. BCTs shown in bold are included in the intervention

b Evidence for effectiveness. Each study reviewed is acknowledged by the following symbols: (✓) positive association; (-) no association; (X) negative association; (blank) BCT not included.

### Stage 2: Refinement and testing of prototype intervention

#### Focus groups and interviews with healthcare professionals

Sixty-five HCPs who deliver prevention services within primary care took part across nine focus groups and two interviews to provide feedback on the prototype intervention. The characteristics of participants are shown in Table 4. Participants included GPs, practice nurses, healthcare assistants, health trainers and managers. Forty-one provided services working for a lifestyle provider and 24 in general practice. The sample included 14 men and 51 women, with varying years of experience in their current working roles. The index of multiple deprivation scores for each of the six general practices were collected (median 12.3, range 9–20.3), five of which were in the highest quintile in the distribution for England. Each of the practices reported that at least 80% (range 79.9–90.7%) of their patient population were of White ethnic origin, followed by at least 6% from Asian ethnic origin (range 6–13.9 %). A small proportion were from other ethnic groups, Black (range 1–2.3%), Mixed (range 1.6–3.5%) and other Non-White (range 0–1.7%).

**Table 4.** Participant characteristics

Participant characteristics	Focus groups/ interviews (*n* = 65)	Usability testing/ online questionnaire (*n* = 22)
Gender		
Male	14	4
Female	51	18
Place of work		
Lifestyle provider	41	12
General Practice	24	10
Job role		
Health Coach/Trainer	33	6
Practice nurse	12	5
General practitioner	7	4
Manager	6	3
Healthcare assistant	3	2
Administrator	3	2
Nutrition student	1	0
Number of years’ experience in this role		
<1 year	17	3
1–2 years	23	9
3–5 years	12	3
5+ years	13	7

Overall participants were enthusiastic and supportive about the intervention and felt that it showed promise for use within primary care consultations and potential to benefit patients and the NHS system as a whole.

*“I think it would help motivate people and actually help them see the bigger picture but also help them take ownership themselves and have that motivation, and seeing where it all connects and what they can do themselves with the right education and support and help.”* (Focus group 3, Lifestyle provider)

*“I would have thought so because obviously anybody that we can prevent or lower their risk of is less work for us and less work for secondary care and less cost to the NHS, and at very little cost to ourselves.”* (Focus group 9, General practice)

We have reported below in turn the results within each of the three constructs of NPT incorporated into the focus group discussions: coherence; cognitive participation; and collective action.

##### Coherence

Within the construct of c*oherence*, which is defined as sense-making, there were several components discussed by participants in each of the focus groups/interviews. All participants could see the importance and benefits of the intervention and the potential value it could have within primary care consultations, especially within the current prevention activities they perform as part of their role. Particular reference was made to the intervention’s potential to act as an additional motivator to behaviour change within other existing conversations about disease risk including CVD.

*“If someone has got high cardiovascular risk and they’ve got a high cancer risk as well…I think if they get all the information in one lump sum they’re more prone to be open to the suggestion of change.”* (Focus group 1, Lifestyle provider)

*“I suppose it’s an additional motivator to reinforce the lifestyle message that you’re trying to give, because you’re not giving them any different advice, you’re still saying, do all the same things in terms of diet and lifestyle.”* (Focus group 5, General Practice)

Many participants were also able to build on their shared experiences of delivered risk information and show understanding of the aims and objectives of the intervention. Visualisation of the change in risk level after a discussion on goal setting for behaviour change was particularly recognised as of value.

*“Definitely think seeing that change, so looking at the risk now, then actually how it can almost be halved if it was going with like the target values that it’s easier for them to visualise that, rather than just being told, “Ah it could reduce”.”* (Focus group 4, Lifestyle provider)

*“I think something interactive is always helpful than just kind of giving information, so something like goal setting…that can definitely help”* (Focus group 2, Lifestyle provider)

This extended to consideration of its delivery, which included the content required to discuss effectively the risk assessment and lifestyle advice with patients.

*“If we only delivered the figure (risk score) to the client, it still remains very abstract to them, so what we need to focus the discussion on is exactly what’s going on and what’s participating to that risk and how we can work with it”* (Focus group 4, Lifestyle provider)

##### Cognitive participation

As part of the discussion, themes related to *cognitive participation*, defined broadly as engagement, were considered. Discussion around this focused on the delivery and incorporation of the intervention. Most participants reported how the delivery of the intervention had the potential to be part of their existing role, and suggested specific procedures that would enable implementation.

*“If a template (electronic) was designed for this then that would be a reminder to us to discuss it. And for the patient it would mean that a lot more information is given and advised and they can take action on their lifestyle and make them aware of it”* (Focus group 10, General Practice)

To ensure that the intervention had the potential to fit within existing practice, discussion also focused on how HCPs could work together to incorporate ways of delivery. Many participants showed willingness to be involved in the delivery of the intervention as part of their role and could see how this could extend to other members of the healthcare team. Most participants recruited from general practice agreed that, after training, practice nurses or healthcare assistants, could deliver the intervention.

*“I think if some training is given I’m sure they’d (Healthcare assistants) be fine, and with our support, nurses’ support, I’m sure they would be able to do that.”* (Focus group 10, General Practice)

##### Collective Action

Participants discussed several aspects of *collective action*, defined as support for delivery, with specific emphasis on the operationalisation of the intervention. Many described availability of resources and integration into existing work within primary care as of importance to its effectiveness.

Within the discussions around resources, most participants agreed that having time available within the consultation was essential to the success of intervention delivery. This included time to explain the risk presentation, discuss lifestyle changes, offer support and answer questions. The time required for completion was felt to be dependent on the individual patients’ personality and level of risk.

*“It depends on the patient. Some people may get really anxious and spend another 10 minutes discussing that, and others will be less anxious and go home. It’s hard to predict.”* (Focus group 5, General Practice)

Alongside time availability, sufficient practitioner training and practical resources were considered by some participants to be important to patient understanding and acceptance of the risk and lifestyle information.

*“We need to have the sufficient training to do that because I know it’s all very well that we sit and we give the information but for them (patients) to fully understand the risks, we need proper training and showing they can reduce the risk but also how we put it across to them. Because it’s got to be a very diplomatic, calm way for them to understand and process the information”* (Focus group 2, Lifestyle provider)

*“Practical problems that we don’t have colour printers and that is very much geared towards the colour.”* (Focus group 7, General practice)

During discussion, many participants went further and evaluated the potential integration of the intervention into their existing work. NHS Health Checks were highlighted as an ideal opportunity for integration as conversations of disease prevention and lifestyle behaviour change are already taking place with patients.

*“If it’s associated with NHS health checks you already get a BMI, the smoking, alcohol and the physical activity as well. And as part of the diet I ask them and normally I type up what they say about diet, if they’re having their five a day (fruit and vegetables) or not at all, and the same with the alcohol. So it’s quite simple and it’s all the questions you’re already asking for the NHS health checks”* (Focus group 10, General practice)

One participant also felt that integration into NHS Health Checks would be received favourably by patients, as many wish to receive comprehensive healthcare from their general practice at each consultation.

*“I think that would be great actually…some patients expect more when they come for their health checks, especially like between 40 s and 60 s when they work and they find it difficult to come for an appointment, they want everything squeezed in that appointment and they would really want to talk more, not just the blood pressure and weight.”* (Focus group 10, General Practice)

However, this was not a universal view with another participant wondering if inclusion into NHS Health Checks would be too much information for the patient to receive in one consultation.

*“I think we just need to be mindful that it may be a little bit heavy for the patient to handle all (CVD, cancer, diabetes, dementia) in one conversation perhaps.”* (Focus group 4, Lifestyle provider)

##### Key feedback and suggestions for improvement

Participants also provided specific feedback and suggestions to improve the intervention. Changes made in response to this included: amendment of the risk presentation to simplify the wording; the option to display risk percentages to enhance interpretation; provision for participants to return to the website to view the risk score and behaviour change advice at a later date; portion sizes chart available to help collection of risk factor information; and inclusion of additional information for signposting to local services and websites. Suggestions that we chose not to incorporate included the possibility to view the risk factor information of the average person rather than the recommended lifestyle guidance, text message reminders of the goals set during the intervention delivery and the option to print in colour. After consideration, it was felt that including additional information about the average person alongside a person of their same age and sex with the recommended lifestyle could be potentially confusing and that adding text message reminders would substantially complicate the delivery, and therefore implementation. It was also not feasible to provide colour printing in practices.

#### Usability testing and feedback from healthcare professionals

Sixty out of the 66 focus group/interview participants agreed to be contacted for participation in the usability testing. Of the 60 invited, 57 provided valid email addresses. Twenty-two of those completed the usability testing and feedback questionnaire (Table 4).

Over 95% felt that collecting the risk factor information and using the website were very easy or easy and that the risk presentation and lifestyle information were very clear or clear. Ninety-five percent also stated that they could use the website in its current form with only 7 of the 22 participants indicating that they would probably or definitely need training. Of those seven, five preferred face-to-face training with a member of the study team, one an online module and one a step-by-step written guide. However, 27% of participants responded that they were unaware of the option of set targets and 5% that they had been unable to set targets.

**Figure 2.**
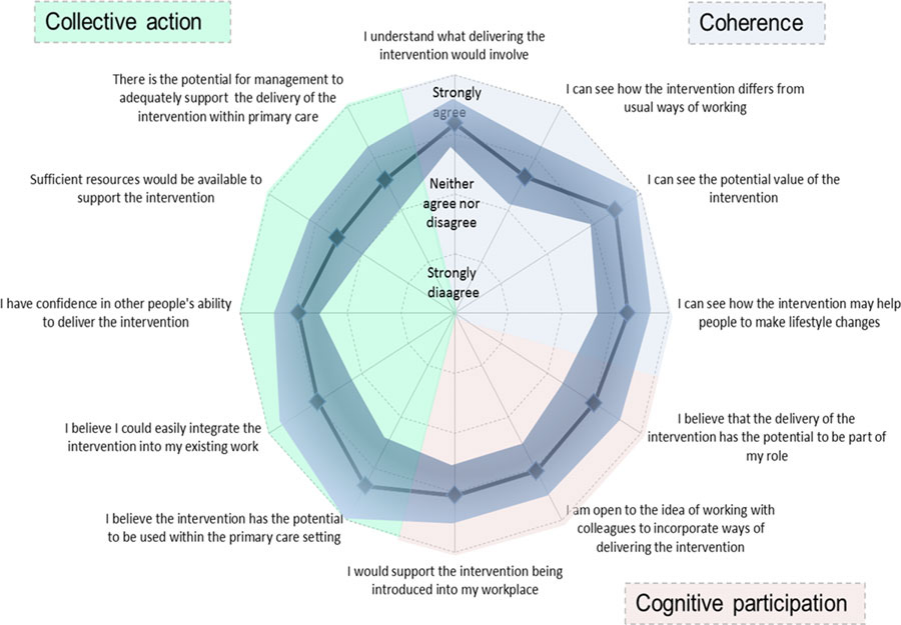
Usability testing results.

Overall, participants felt the intervention had the potential to become a normal part of their work [mean score 8.0 (SD 1.5, *n* = 21) on a scale from 1 (not at all) to 10 (completely)]. Figure 2 shows a summary of the mean responses to individual questions addressing coherence, cognitive participation and collective action. The highest scores reported [mean score 4.45 (SD 0.49), *n* = 21) on a scale from 1 (strongly disagree) to 5 (strongly agree)] indicated participants agreed/strongly agreed that they could see the potential value of the intervention and more specifically its use in the primary care setting [mean score 4.33 (SD 0.89), *n* = 20]. In contrast, lower scores were reported by participants when on considering if the intervention differed from usual ways of working [mean score 3.63 (SD 0.56), *n* = 22]. Confidence in others to deliver the intervention [mean score 3.90 (SD 0.41), *n* = 22] and belief that the intervention could easily integrate into existing work [mean score 3.94 (SD 0.75), *n* = 19] were also reported with moderate agreement by participants.

When asked specifically whether they believed the intervention could easily be integrated into practice, over 90% (*n* = 21) of respondents strongly agreed or agreed that it could easily integrate into NHS Health Checks, chronic disease reviews or lifestyle advice sessions. Fewer (74%, *n* = 19), however, strongly agreed or agreed that it could easily integrate into routine practice, with five (26%, *n* = 19) neither agreeing nor disagreeing. Consistent with the lower scores in the collective action domains regarding sufficient resources [mean score 3.52 (SD 0.58)] and potential for management to adequately support the delivery of the intervention [mean score 3.57 (SD 0.59)], only eight (38%, *n* = 21) agreed that the intervention could be delivered within 5 min, with five (24 %, *n* = 21) neither agreeing nor disagreeing and eight (38 %, *n* = 21) either disagreeing or strongly disagreeing.

##### Key feedback and suggestions for improvements

In response to the difficulties some HCPs had setting targets, we changed the layout and some of the text on the website to make this step clearer. Participants also provided further suggestions for refinement of the intervention in the free text questions following the usability testing. These included changes to the units of measurement for calculation of alcohol consumption and body mass index and the option to print individual pages of the lifestyle leaflet to support specific goals.

In response to the feedback gathered from the HCPs on aspects of training, we also devised a face-to-face training package, which could be delivered by the study team and included the opportunity to simulate delivery of the intervention on the website to gain familiarity.

## Discussion

### Key findings

In this paper, we have described the development of a very brief intervention to deliver personalised cancer risk information in primary care and demonstrated the value of integrating theory- and evidence-based approaches with primary data collection in that process. Using the NPT framework prospectively to guide the overall format of the intervention and behaviour change theory and published literature to guide the content, we were able to systematically identify key implementation considerations at the design stage and select risk presentation formats and BCTs associated with changes in the target behaviours, increasing the potential both for future incorporation of the intervention into practice and intervention effectiveness (Baker *et al.*, 2010; Glanz and Bishop, 2010). Including qualitative data collection with HCPs involved in delivering prevention activities within primary care throughout the process further allowed us to rehearse the prototype intervention with those who will be delivering it and refine the intervention in response to their comments. Feedback on the initial prototype suggested support and enthusiasm for its use, highlighting its potential benefit to patients, especially acting as an additional motivator to behaviour change within other current conversations of risk in primary care, namely in NHS Health Checks. Feedback on the intervention and the results of usability testing indicated that HCPs found the intervention to be acceptable, understood its purpose, and believed that it had the potential for implementation into primary care consultations. They could also see the potential value of the intervention and its ability to promote lifestyle changes. However, they remained concerned about whether sufficient time, resources and support would be available.

A particular strength and novel aspect of our approach is the use of NPT prospectively as a framework when considering the overall format of the intervention. In a recent systematic review of the use of NPT in feasibility studies and process evaluations (May *et al.*, 2018), only one published study has used NPT prospectively in the intervention development phase of a study (Brooks *et al.*, 2015). We chose NPT because it focuses on understanding how and whether complex interventions become routinely embedded in healthcare practice (May *et al.*, 2009). This includes components relevant to both the individual and the context in which the intervention will be delivered. This was important as we had identified from previous research with HCPs that the main barriers to discussing cancer risk in practice included individual concerns about understanding and communicating risk and context-specific needs for time and resources (May *et al.*, 2009). While there are other approaches we could have applied, such as intervention mapping (Bartholomew *et al.*, 1998) and the Consolidated Framework for Implementation Research (CFIR) (Damschroder *et al.*, 2009), the accompanying NoMAD checklist also provided key questions through which we could obtain feedback from HCPs across the first three domains of NPT.

This feedback was important. At a time when both workload is increasing and funding is decreasing, the engagement of those working within primary care is more important than ever. Complexity science has also shown that in complex adaptive systems, such as healthcare (Braithwaite *et al.*, 2018), professionals tend to accept new ideas based on their own logic rather than the views of others, and are more likely to accept change when they are involved in the process than when change is imposed on them by others (Braithwaite, 2018). Engaging with HCPs at an early stage in the intervention development process therefore allowed us to incorporate the views of professionals who would ultimately deliver the intervention and maximise the likelihood of future incorporation in practice. Consistent with the concept of intervention plasticity within NPT, and analogous to the distinction between the ‘core components’ and the ‘adaptable periphery’ described with the CFIR (Damschroder *et al.*, 2009), we also did not attempt to develop a standardised process for the delivery of the intervention. Instead, we consider the intervention as a set of tools which HCPs can adapt to different consultations and patient groups. For example, in an NHS Health Check, the HCPs may choose to complete the risk assessment and risk communication elements alongside the assessment and communication of CVD risk and then discuss the risk management advice for both cancer and CVD together, or may choose to separate discussions about CVD and cancer within the consultation.

The overall enthusiasm we found amongst these HCPs for the intervention mirrors that seen in other studies which have found that primary care HCPs consider prevention activities an important aspect of their role (Brotons *et al.*, 2005; McIlfatrick *et al.*, 2013, 2014; Usher-smith *et al.*, 2017a). As in this study, many also believed patients wanted to change and would follow their recommendations, although belief was higher amongst practice nurses (McIlfatrick *et al.*, 2014) than GPs (McIlfatrick *et al.*, 2013). The concerns about time and resources are also consistent with previous research (Brotons *et al.*, 2005; McIlfatrick *et al.*, 2013; Usher-smith *et al.*, 2017a). This is despite our aim to develop an intervention that would be very brief and limit the additional resources required, highlighting the challenges of developing interventions that are likely to be both effective and widely used.

Our use of behaviour change theory, reviews of existing evidence in the literature and expert opinion to guide the development of the content of the intervention further enabled us to maximise the potential effectiveness. However, our approach has its limitations. Firstly, when assessing the effectiveness of BCTs, we used evidence from systematic reviews in which meta-regression had been used to identify which BCTs were more effective for achieving change in a given behaviour. The use of meta-regression with study-level information to make inferences about individual-level change relies on indirect comparisons and so is at risk of ecological fallacy or aggregation bias. The relationships between BCTs and behaviour change seen in these reviews may therefore not reflect the relationships between individual BCTs and behaviour change in experimental studies. Most of the evidence on effectiveness of BCTs also relate to individual behaviours, such increasing physical activity while our intervention targets multiple behaviours.

Secondly, although we purposefully recruited a diverse range of HCPs with different roles and years of experience from both general practice and lifestyle provider services, most general practices were from areas of low deprivation, with patients predominantly of White ethnic origin. The views of the HCPs in this study may, therefore, not reflect the views of those working in areas of higher deprivation or different ethnic backgrounds where there may be additional pressures on HCP time, language barriers, or differences in patient understanding and beliefs. We also acknowledge that the professionals who took part may have self-selected due to positive views about health promotion.

We also took examples of the components of the prototype intervention to the focus groups and interviews. While this provided a springboard for discussion and we were able to collect both positive and negative feedback on our prototype versions, it may have made it harder for participants to consider what was really important to them and they may have been more reluctant to voice contradictory opinions.

Thirdly, we chose to focus on the views of HCPs rather than patients. While this meant we did not include feedback directly from patients on the intervention during this developmental stage, we did consider the patient perspective throughout the process. This included working closely with our two patient and public representatives, considering patient views within the wider literature and previous qualitative work with patients on the provision of risk-based cancer information (Usher-Smith *et al.*, 2017b). Patient feedback will be a central component of future work piloting the intervention.

Although not necessarily limitations, the iterative nature of the intervention development also brought with it a number of challenges. Involving over 60 HCPs in the process meant we heard multiple, and in some cases conflicting, perspectives on the intervention and received a large number of suggestions for changes. In some cases, the decision to implement a change or not was straightforward. These included changes that were limited by practical constraints, such as the suggestion to print the patient information in colour within the consultation, and features that the HCPs consistently thought would be difficult to implement, such as assessing daily rather than weekly alcohol intake. At other times, however, it was a challenge to decide when to implement a change based on their feedback and when not to. For example, we chose not to include the possibility to view the risk factor information of the average person rather than the recommended lifestyle guidance. In these cases, we tried to balance what the majority of participants would benefit from as a reference point.

The potential time and cost of multiple iterations of changes to a digital intervention was also a challenge as we were working with a computer programming team to develop the website (Yardley *et al.*, 2015). We addressed this by developing a close collaboration with the programmers from the start and arranged for them to train one of the research team to make minor alterations without needing to go back to them each time. We also took screenshots of potential pages from the intervention to the early focus groups and interviews rather than developing the website at that stage. Despite this though, the process was time-consuming and the potential risk of overspend significant so it is an important consideration for others developing digital interventions.

## Conclusions

In conclusion, we have described how using NPT prospectively alongside behaviour change theory and reviews of the published literature can be successfully used to develop an evidence-based personalised cancer risk-based intervention to provide information and promote behaviour change in primary care. HCPs involved in the delivery of prevention activities welcomed the intervention and provided essential feedback for its refinement. The next step is to pilot the intervention with patients and HCPs within primary care consultations. Recognising that implementation is an ongoing iterative process rather than a linear one (Damschroder *et al.*, 2009), a key element of that evaluation will be working with HCPs to help them adapt the intervention to their practice. Central to supporting that process and preparing for the future scaling up of the intervention will also be an evaluation of the potential unintended consequences of the intervention and developing ways of working with HCPs to support them to overcome implementation challenges (Paina and Peters, 2012).

## Data Availability

All the data will be stored in accordance with the Data Protection Act 1998 within the University of Cambridge data repository (https://www.repository.cam.ac.uk/) for at least 10 years from the last access. All anonymised data will be publicly available via that repository with links. Focus group and interview transcripts containing pseudo-anonymised data will be stored in the repository, and formal requests for access will be considered via a data sharing agreement that indicates the criteria for data access and conditions for research use and will incorporate privacy and confidentiality standards to ensure data security.

## Supplementary material

For supplementary material accompanying this paper visit https://doi.org/10.1017/S146342361900080X. click here to view supplementary material
