# Supplementary material for: Development and usability testing of a very brief intervention for personalised cancer risk assessment to promote behaviour change in primary care using normalisation process theory
Source: Prim Health Care Res Dev. 2020 Jan 14;21:e1. doi: 10.1017/S146342361900080X (PMC7005588; doi:10.1017/S146342361900080X)
Supplement: Supplementary file 1 [file S146342361900080Xsup001.pdf]

UNIVERSITY OF CAMBRIDGE

Funded by CANCER RESEARCH UK

I-CaPP

Interventions for Cancer Prevention in Primary Care

Please tick **each** box

I confirm that I read and understood the participant information leaflet (version 1, 24th March 2017) before participating in the focus group/interview. I have had the chance to think about the information and contact the study team to ask questions.	☐
I understand that my participation is voluntary and that I am free to withdraw at any time, without giving any reason.	☐
I understand that anonymous direct comments from this questionnaire may be published in journals or presented at conferences, but without my name or other identifying details used, I give permission for my comments to be used for that purpose.	☐

By clicking 'I agree' below you are indicating that you have read and understood this consent form, and agree to participate in this research study.

I agree

This survey is designed to gain a better understanding of the most effective ways to discuss cancer risk and prevention in primary care.

We understand that people have different roles, and that people may have more than one role.

From the statements below please choose an option that best describes ***your main role*** in relation to the prevention services*:*

☐ **I am involved in managing or overseeing prevention services e.g. NHS Health Checks**

☐ **I am involved in delivering prevention services e.g. NHS Health Checks**

☐ **I am involved in commissioning prevention services e.g. NHS Health Checks**

For this survey, please answer all the statements from the perspective of this role. Depending on your role or responsibilities, some statements may be more relevant than others.

You will be prompted to look at each intervention in turn on the screen. This will remain open in another window while you complete the survey.

For each intervention you will complete 2 parts.

Part A includes two general questions about the intervention.

Part B contains a set of more detailed questions about the intervention. For each statement in Part C, there is the option to agree or disagree with what is being asked **(Option A).** However, if you feel that the statement is not relevant to you, there are also options to tell us why **(Option B).**

The final part to the survey, **Part C** asks some brief questions about yourself and your role.

There is also a comments box at the end of the questionnaire if you would like to share additional thoughts about any of the interventions.

Please take the time to decide which answer **best suits your experience for each statement and tick the appropriate option.**

PLEASE LOOK AT INTERVENTION 1

Part A: General questions about intervention 1

1. Now that you have had the opportunity to view the intervention, how familiar does it feel?

Still feels very new					Somewhat					Feels completely familiar
	1	2	3	4	5	6	7	8	9	10

2. Do you feel the intervention has the potential to become a normal part of your work?

Not at all					Somewhat					Completely
**0**	1	2	3	4	5	6	7	8	9	10

Part B: Detailed questions about intervention 1

For each statement please select an answer that best suits your initial thoughts.

Section B1	Strongly agree	Agree	Neither agree nor disagree	Disagree	Strongly disagree
1. I understand what delivering the intervention would involve	O	O	O	O	O
2. I can see how the intervention differs from usual ways of working	O	O	O	O	O
3. I can see the potential value of the intervention	O	O	O	O	O
4. I can see how the intervention might help people to make lifestyle changes	O	O	O	O	O

For each statement please select an answer that best suits your initial thoughts using **Option A**. If the statement is **not** relevant to you please select **Option B**.

	Option A					Option B
Section B2	Strongly agree	Agree	Neither agree nor disagree	Disagree	Strongly disagree	Not relevant to my role
1. I believe that the delivery of the intervention has the potential to be part of my role	O	O	O	O	O	O
2. I'm open to the idea of working with colleagues to incorporate ways of delivering the intervention	O	O	O	O	O	O
3. I would support the intervention being introduced into my workplace/role	O	O	O	O	O	O

For each statement please select an answer that best suits your initial thoughts using **Option A**. If the statement is **not** relevant to you please select **Option B**.

Section B3	Option A					Option B
	Strongly agree	Agree	Neither agree nor disagree	Disagree	Strongly disagree	Not relevant to my role
1. I believe the intervention has the potential to be used within the primary care setting	○	○	○	○	○	○
2. I believe I could easily integrate the intervention into my existing work	○	○	○	○	○	○
I believe the intervention could easily integrate into…<br>3a) NHS Health Checks	○	○	○	○	○	○
3b) Chronic disease reviews	○	○	○	○	○	○
3c) routine consultations	○	○	○	○	○	○
3d) [other to be defined after focus groups]	○	○	○	○	○	○
4. I believe the intervention could be delivered within 5 minutes	○	○	○	○	○	○
5. I have confidence in other people's ability to deliver the intervention	○	○	○	○	○	○
6. Sufficient resources would be available to support the intervention	○	○	○	○	○	○
7. There is the potential for management to adequately support the delivery of the intervention within primary care	○	○	○	○	○	○

NOW PLEASE LOOK AT INTERVENTION 2

Part A: General questions about intervention 2

1. Now that you have had the opportunity to view the intervention, how familiar does it feel?

Still feels very new					Somewhat					Feels completely familiar
**0**	1	2	3	4	5	6	7	8	9	10

2. Do you feel the intervention has the potential to become a normal part of your work?

Not at all					Somewhat					Completely
**0**	1	2	3	4	5	6	7	8	9	10

Part B: Detailed questions about intervention 2

For each statement please select an answer that best suits your initial thoughts.

Section B1	Strongly agree	Agree	Neither agree nor disagree	Disagree	Strongly disagree
1. I understand what delivering the intervention would involve	○	○	○	○	○
2. I can see how the intervention differs from usual ways of working	○	○	○	○	○
3. I can see the potential value of the intervention	○	○	○	○	○
4. I can see how the intervention might help people to make lifestyle changes	○	○	○	○	○

For each statement please select an answer that best suits your initial thoughts using **Option A**. If the statement is **<u>not</u>** relevant to you please select an answer from **Option B**.

	Option A					Option B
Section B2	Strongly agree	Agree	Neither agree nor disagree	Disagree	Strongly disagree	Not relevant to my role
1. I believe that the delivery of the intervention has the potential to be part of my role	○	○	○	○	○	○
2. I'm open to the idea of working with colleagues to incorporate ways of delivering the intervention	○	○	○	○	○	○
3. I would support the intervention being introduced into my workplace/role	○	○	○	○	○	○

For each statement please select an answer that best suits your experience using **Option A**. If the statement is **<u>not</u>** relevant to you please select an answer from **Option B**.

Section B3	Option A<br>Strongly agree	Agree	Neither agree nor disagree	Disagree	Strongly disagree	Option B<br>Not relevant to my role
1. I believe the intervention has the potential to be used within the primary care setting	O	O	O	O	O	O
2. I believe I could easily integrate the intervention into my existing work	O	O	O	O	O	O
I believe the intervention could easily integrate into…<br>3a) NHS Health Checks	O	O	O	O	O	O
3b) Chronic disease reviews	O	O	O	O	O	O
3c) routine consultations	O	O	O	O	O	O
3d) [other to be defined after focus groups]	O	O	O	O	O	O
4. I believe the intervention could be delivered within 5 minutes	O	O	O	O	O	O
5. I have confidence in other people's ability to deliver the intervention	O	O	O	O	O	O
6. Sufficient resources would be available to support the intervention	O	O	O	O	O	O
7. There is the potential for management to adequately support the delivery of the intervention within primary care	O	O	O	O	O	O

Part C: About yourself

1. How would you describe your professional job category?

- ☐ Health trainer
- ☐ GP
- ☐ Practice nurse
- ☐ Health care assistant
- ☐ Health coach
- ☐ Manager of lifestyle services
- ☐ Public Health professional
- ☐ Other, please specify

2. How many years have you worked in this role?

- ☐ Less than one year
- ☐ 1-2 years
- ☐ 2-3 years
- ☐ 3-5 years
- ☐ 6-10 years
- ☐ 11-15 year
- ☐ More than 15 years

Additional comments

Please include any additional thoughts about the intervention here.

Thank you for completing our survey.
